# Effects of Oral Inflammatory Diseases and Oral Hygiene on Atrial Fibrillation: A Systematic Review

**DOI:** 10.1155/2023/1750981

**Published:** 2023-03-27

**Authors:** Zhanxin Zhang, Fei Chen, Xueying Gao, Bing Xiao, Fan Liu, Jingchao Lu

**Affiliations:** The Second Department of Cardiovascular Medicine, The Second Hospital of Hebei Medical University, Shijiazhuang 050000, China

## Abstract

**Objective:**

Research evidence suggests a link between periodontitis (PD) and atrial fibrillation, but the nature of this link is unclear. This study aimed to systematically review and evaluate the association between PD, other oral diseases, and atrial fibrillation and the role of oral hygiene in preventing atrial fibrillation.

**Methods:**

We searched the Medline, Embase, Cochrane Library, and Web of Science databases for the clinical study of oral health and atrial fibrillation from inception to November 2022. Oral health conditions included PD and other oral inflammatory diseases, regular oral hygiene, and tooth brushing. The primary outcomes were the risk of new-onset atrial fibrillation in patients with oral disease, the effect of regular oral care on preventing atrial fibrillation, the effect of frequent tooth brushing on preventing atrial fibrillation, and the incidence of atrial fibrillation in PD patients.

**Results:**

Eight clinical trials with a total of 4,328,355 patients were included. The result of the research showed that PD and other impaired oral health may be associated with new-onset atrial fibrillation, and its severity was dose-responsive to the risk of atrial fibrillation. The incidence of atrial fibrillation in patients with severe PD was about 16.3%. Moreover, PD may increase the risk of long-term arrhythmia in patients with atrial fibrillation. Regular oral care and frequent tooth brushing can reduce the incidence of atrial fibrillation.

**Conclusion:**

Regular and moderate oral hygiene, frequent tooth brushing, and prevention of PD and other oral inflammatory diseases could reduce the occurrence of atrial fibrillation. It is recommended to strengthen the popularization of oral health knowledge in the publicity related to atrial fibrillation.

## 1. Introduction

Atrial fibrillation (AF) is a common tachyarrhythmia in clinical practice that can increase the risk of cardiovascular and cerebrovascular diseases such as stroke and heart failure and is an important public health problem [1, 2]. Based on aging populations worldwide and the fact that the success rate of atrial fibrillation ablation still needs to be improved [3], how to effectively prevent the occurrence of atrial fibrillation has always been a key concern.

Multiple studies have shown that inflammation could promote electrical remodeling and structural remodeling of the atrium, which plays an important role in the occurrence and development of atrial fibrillation [4, 5]. In addition, C-reactive protein, interleukin-6, tumor necrosis factor-*α*, and other inflammatory factors can cause an abnormal electrical activity of pulmonary veins, shorten atrial action potential, and interact with heat shock protein or myeloperoxidase to promote atrial fibrosis [6], thereby promoting the occurrence and recurrence of atrial fibrillation and thromboembolic events.

Oral inflammatory diseases are evolving chronic diseases. Periodontitis (PD) is a major oral health problem, leading to tooth loss and bacteremia and resulting in systemic inflammatory responses in severe cases. Surveys show that approximately 50% of the world's population suffers from PD and 10% suffer from severe PD, which is considered to be the sixth global epidemic affecting every country [7, 8]. Based on common risk factors and underlying pathophysiological mechanisms, increasing attention has been given to the association between oral diseases and cardiovascular diseases. Studies have shown that in PD patients, *Porphyromonas gingivalis* and inflammatory factors could promote the progression of atherosclerosis and may be potential risk factors for coronary heart disease [9]. PD can increase the risk of hypertension through systemic inflammation and oxidative stress [10, 11]. Subsequently, related studies on oral inflammatory diseases such as PD and atrial fibrillation have gradually increased. However, there is no consensus on the specific relationship between oral inflammatory diseases and atrial fibrillation and whether oral care can lessen the risk of atrial fibrillation. Therefore, this study analyzed relevant clinical trials published thus far and conducted a systematic review to analyze the effects of oral inflammatory diseases and oral hygiene on atrial fibrillation.

## 2. Materials and Methods

### 2.1. Data Source and Search

Clinical studies related to oral health and atrial fibrillation published in Medline, Embase, Cochrane Library, and Web of Science were searched. The retrieval time was from the establishment of each database to November 2022. There was no restriction on the language of the included literature, and we attempted to translate non-English literature (if not available, the literature was excluded). Each database was searched in detail using Medical Subject Headings (MeSH) terms and associated free words. The search keywords included “Atrial Fibrillation” and its related free words, “Periodontitis” and its related free words, “atrial flutter,” “atrial tachycardia,” “atrial arrhythmia,” “periodontal disease,” “pulpitis,” “pericoronitis,” “periapical,” “dental abscess,” “tooth abscess,” “endodontic abscess,” “pulpal abscess,” “apical abscess,” “periradicular abscess,” “radicular abscess,” and “acute dental infection.” Supplementary Table S1 summarizes the specific retrieval strategies and results used for each database. In addition, in order to obtain more relevant studies, a manual search was conducted for references that might be included in the literature. This study was conducted according to the PRISMA (Preferred Reporting Items for Systematic Reviews and Meta-Analysis) guidelines and statement.

### 2.2. Inclusion/Exclusion Criteria

The inclusion criteria were as follows:

1. Prospective or retrospective studies (randomized controlled trials, cohort studies, case-control studies, and cross-sectional studies) on the relationship between oral health and atrial fibrillation in adults (≥18 years of age)
2. Oral health conditions include oral inflammatory diseases such as PD and dental periapical abscess and oral care such as dental cleaning and frequent tooth brushing
3. Outcome indicators were new-onset or recurrent atrial fibrillation
4. Complete baseline data and outcome indicators were available

The exclusion criteria were as follows:

Studies with incomplete data, meta-analyses, reviews, reviews of the literature, case reports, conference abstracts, and other types of literature, and the full text of the literature could not be obtained. When duplicate studies were found, the study with relatively complete data was selected and analyzed. Two researchers independently searched the literature, extracted data, and cross-checked the data. Disagreements were resolved through discussion and negotiation, and remaining disagreements were resolved by a senior third person.

### 2.3. Data Extraction and Evaluation Indicators

The following information was extracted for each included study: general information, including title, first author, journal, and publication year; the country or city where the study was conducted; research characteristics, including study design, sample size, average age, sex ratio, oral health situation, and so on; and study endpoint events (such as the incidence of atrial fibrillation). Follow-up times were extracted from cohort studies and randomized controlled clinical trials.

Evaluation indicators included association between oral inflammatory disease and the risk of atrial fibrillation in univariate and multivariate analyses, such as the hazard ratio (HR), relative risk (RR), odds ratio (OR), and 95% confidence intervals (95% CI); the effect of frequent tooth brushing on atrial fibrillation in multivariate analysis; the effect of regular oral care on atrial fibrillation in multivariate analysis; the incidence of atrial fibrillation in patients with or without PD; and the prevalence of PD in patients with or without atrial fibrillation.

### 2.4. Literature Quality Evaluation

The quality of the included studies was evaluated independently by two researchers. For the included observational studies, the NOS (Newcastle–Ottawa scale, range 0–9) was used to evaluate the research quality. This scale includes 3 major sections (cohort studies include subject selection, comparability between groups, and outcome measurement; case-control studies include subject selection, comparability between groups, and measurement of exposure factors), with a total of 8 item evaluations and a full score of 9 points. A study with an NOS score ≥6 was considered a high-quality study. The quality of the included randomized controlled trials (RCTs) was assessed using the RCT bias risk assessment tool recommended by the Cochrane Handbook for Systematic Reviews 5.1. Disagreements were resolved by consensus. When there is a disagreement, it should be resolved by consensus, and remaining discrepancies could be resolved by a third senior researcher.

### 2.5. Heterogeneity and Statistical Analysis

Heterogeneity generally includes clinical heterogeneity, methodological heterogeneity, and statistical heterogeneity. Clinical heterogeneity includes differences in patient, interventions, control, outcome, and study design. Methodological heterogeneity includes study type, blind method, completeness of outcome report, and rigor of statistical method. When data could be meta-analyzed, *Q* test and *I*^2^ value were usually used to evaluate the statistical heterogeneity. If *I*^2^ > 50%, there was heterogeneity among the studies, and a random effects model was used to analyze the data; if *I*^2^ ≤ 50%, the studies were homogenous, and a fixed effects model was used for analysis. When the data met the requirements, metaregression and subgroup analysis were used to further evaluate the source of heterogeneity, so as to explore the influence of a factor on the effect size. Potential sources of heterogeneity included age, sex, country, study design, and sample size. In addition, sensitivity analysis was attempted to ensure the stability of the results and to analyze the sources of heterogeneity, which was usually conducted by excluding studies sequentially. The sensitivity analysis referred to the pooled analysis of the remaining literature after the removal of a study, the comparison of the combined effect size before and after the removal, to further explore the impact of the excluded studies on the pooled effect size, and to find the source of heterogeneity. If there was no significant change in the pooled effect size after deleting a study, it indicated that the results of meta-analysis were relatively stable; on the contrary, it indicates that the stability of the meta-analysis was poor.

When the data met the requirements, Review Manager statistical software V5.3 was used for data processing and meta-analysis for each outcome index. All evaluation indexes in the included literature were analyzed. The RR, OR, or HR was used as the effect index for categorical variables, and weighted mean square deviation (WMD) was used as the effect index for continuous variables, which were expressed with 95% CIs. *P* < 0.05 was considered statistically significant. When the heterogeneity was too obvious and cannot be resolved, meta-analysis should be abandoned and only systematic review should be conducted.

### 2.6. Publication Bias

Large publication bias may affect the true result of the study. When the number of included studies was more than 10 papers, Egger's test and Begg's test in STATA statistical software V16.0 were used to draw a funnel plot to test for publication bias. Publication bias exists if the *P* values from the tests are all less than 0.1. When the test results of the two groups were inconsistent, considering that the previous studies posited that Egger's test was more sensitive than Begg's test, and the results obtained by Egger's test were selected.

### 2.7. Ethical Approval Statement

Institutional review board approval was not required because the analysis was based on the secondary processing of data from previously published studies.

## 3. Results

### 3.1. Literature Search and Screening Results

**Figure 1:**
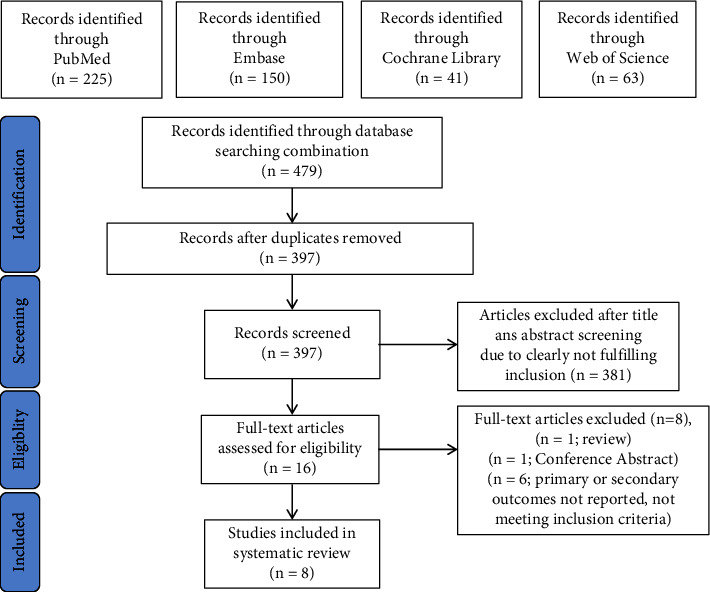
Flow diagram of the literature search and the article evaluation process.

A total of 479 studies were retrieved, of which 397 articles remained after 82 duplicate articles were removed. During the preliminary screening, two researchers independently read the title, abstract, and keywords. After excluding the irrelevant literature and other types of literature, such as reviews, comment, guideline, letter, meta-analysis, animal experiment, and case reports, 16 eligible studies were obtained. After in-depth reading of the full text, 8 studies were excluded according to the data evaluation results, and 8 studies were ultimately included [12–19]. The specific process is shown in Figure 1.

### 3.2. Basic Characteristics and Quality Evaluation of the Included Literature

The eight included articles were observational studies, one of which was a large prospective cohort study [12], five were large retrospective cohort studies based on a national population [13–17], one was a case-control study [18], and one was a cross-sectional study [19]. A total of 4,328,355 patients were included. All six cohort studies included patients without a history of atrial fibrillation at the baseline. Four of the studies included PD patients and non-PD patients in the exposed and nonexposed groups, respectively [12–14, 17]; one study included patients with apical abscesses and nonapical abscesses [15]; and one study included patients with regular oral hygiene and those without oral hygiene [16]. The outcome measure in the above mentioned studies was differences in new-onset atrial fibrillation. Two groups of patients (nonvalvular atrial fibrillation with PD and nonvalvular atrial fibrillation without PD) were included in the case-control study, and the outcome indicators were the effects of periodontitis on arrhythmia events and major adverse cardiac events in patients with atrial fibrillation [18]. The sample sizes of the abovementioned study population ranged from 227 to 3,056,291, with follow-up periods ranging from 18 months to 17 years. A cross-sectional study, including 5,634 participants with complete data on periodontitis and atrial fibrillation, was conducted to assess the relationship between periodontitis and its severity and atrial fibrillation [19]. Specific baseline data included in the literature are shown in Table 1.

Seven observational studies other than cross-sectional studies were assessed for quality based on NOS scores. In the case-control study by Im et al. [18], NOS scores based on the case-control study were used (scoring categories included selection, comparability, and exposure). Because of confounding factors such as age were not matched between the case group and the control group, the NOS score of this study was 7. For cohort studies, NOS scores based on the cohort study were used (scoring categories included selection, comparability, and outcome). NOS scores ranged from 7 to 9, indicating high quality of the included literature. The kappa value of agreement between the two researchers at the quality evaluation stage was 0.73 (95% confidence interval [CI]: 0.38–1.00), indicating a high consistency (*P* < 0.001). Details of the quality scores for each included study are presented in Table 2.

### 3.3. Association between Oral Health and Atrial Fibrillation

#### 3.3.1. Effects of Oral Inflammatory Disease on Atrial Fibrillation

The effect of oral inflammatory disease on the risk of atrial fibrillation was reported in seven studies [12–15, 18]. Four of them analyzed the correlation between PD and atrial fibrillation. A retrospective cohort study [14] by Chen et al. found that PD patients had a significantly increased risk of new-onset atrial fibrillation/flutter after adjusting for confounders (HR 1.31, 95% CI 1.25–1.36). A case-control study [18] by Im et al. reported that PD was an independent predictor of major adverse cardiac events (OR 17.78, 95% CI 3.46–91.34) and arrhythmic events (OR 9.19, 95% CI 1.24–67.96) after adjusting for the confounders' factor. In a retrospective cohort study [13] by Chang et al., univariate analysis suggested that PD increased the risk of new-onset atrial fibrillation (HR 1.1, 95% CI 1.02–1.20), but multivariate analysis showed no significant association between PD and atrial fibrillation. Hsu et al. [15] investigated the effect of PD on stroke and found that patients with PD had a significantly increased risk of AF compared with patients without PD (OR 1.39, 95% CI 1.30–1.48), but the study did not adjust for the influence of confounding factors. After adjusting for confounding factors, the incidence of stroke in PD patients was 2.14 times that of non-PD patients. Both studies of Sen et al. (2021) and Struppek et al. (2021) included patients with different degrees of PD and only provided the risk of atrial fibrillation in different degrees of PD after adjusting for confounding factors and did not provide the total risk of atrial fibrillation in all the PD patients [12, 19]. One article analyzed the association between dental periapical abscess and atrial fibrillation. Multivariate analysis of retrospective cohort studies [15] by Hassan et al. showed that dental periapical abscess was significantly associated with new-onset atrial fibrillation (HR 1.11, 95% CI 1.01–1.22).

#### 3.3.2. Effects of Different Degrees of Periodontitis on Atrial Fibrillation

Two studies reported the different effects of PD severity on the occurrence risk of atrial fibrillation [12, 19], involving healthy patients and mild PD patients (4,588 patients), moderate PD patients (5,197 patients), and severe PD patients (2,382 patients). The prospective cohort study [12] by Sen et al. included healthy individuals, mild PD, moderate PD, and severe PD patients. Univariate analysis found that the severity of periodontitis was associated with a dose-response relationship with atrial fibrillation, but only severe PD significantly increased the risk of new-onset AF (HR 1.31, 95% CI 1.06–1.62) after adjusting for confounding factors. Struppek et al. [19] included healthy/mild PD, moderate PD, and severe PD patients in a cross-sectional study, and only severe PD was associated with new-onset atrial fibrillation (OR 1.66, 95% CI 1.16–2.36) in univariate analysis, and there was no significant correlation between various degrees of periodontitis and atrial fibrillation after adjusting for confounding factors.

#### 3.3.3. Incidence of Atrial Fibrillation in Patients with Oral Inflammatory Disease

Four studies [12–15] reported the incidence of atrial fibrillation in patients with and without oral inflammatory disease, among which three cohort studies [12–14] showed that the incidence of atrial fibrillation in PD patients was significantly higher than that in non-PD patients (Chang Y study: 3.29% *vs.* 3.01%; Sen S study: 14.39% *vs.* 11.80%; and Chen DY study: 2.07% *vs.* 1.57%). A retrospective cohort study [15] showed that the incidence of atrial fibrillation in patients with apical abscess was significantly lower than that in patients without apical abscess (9.27% *vs.* 10.69%), but apical abscess was an independent predictor of new-onset atrial fibrillation after adjusting for confounders.

In addition, a prospective cohort study [12] showed that the incidence of atrial fibrillation in healthy individuals, mild PD, moderate PD, and severe PD patients was 11.8%, 11.9%, 15.3, and 16.3%, respectively. A cross-sectional study [18] showed a higher prevalence of atrial fibrillation in men than in women among patients with severe PD at the same age, and the difference was particularly significant in older patients >65 years (29.1% *vs.* 19.7%), which has not been explicitly reported in other studies.

#### 3.3.4. Prevalence of Periodontitis in Patients with Atrial Fibrillation

A case-control study [18] by Im et al. reported the prevalence of PD in patients with atrial fibrillation; 47 of 227 (20.7%) patients with atrial fibrillation had PD. Atrial fibrillation patients with PD and without PD were followed up for an average of approximately 18 months, and arrhythmia events in PD patients were significantly higher than those in patients without PD (93.6% *vs.* 17.4%, *P* < 0.001). Multivariate analysis suggested that PD was an independent risk factor for arrhythmia and major adverse cardiac events during follow-up in patients with atrial fibrillation.

#### 3.3.5. Effects of Regular Oral Care on Atrial Fibrillation

Four studies [12–14, 16] reported the effect of oral care on the occurrence risk of atrial fibrillation, among which two reported the positive effect of regular oral care in reducing the risk of atrial fibrillation [12, 16]. Multivariate analysis by Chen et al. [16] suggested that dental scaling at least once a year for 3 consecutive years was associated with a lower risk of new-onset atrial fibrillation (HR 0.67, 95% CI 0.52–0.86). Univariate and multivariate analyses by Sen et al. [12] both indicated that compared with episodic users, regular dental care had a significantly lower risk of new-onset atrial fibrillation (adjusted HR 0.88, 95% CI 0.78–0.99). However, a univariate analysis by Chang et al. [13] found that regular professional dental cleaning significantly reduced the risk of atrial fibrillation (HR 0.87, 95%CI 0.81–0.93), but multivariate analysis did not suggest a significant association.

Chen et al.'s study [14] reported that dental cleanings 0–2/year were a protective factor for atrial fibrillation (HR 0.39, 95% CI 0.38–0.41), but dental cleanings >2 times per year were a risk factor for atrial fibrillation (HR 6.06, 95% CI 5.38–6.83). However, this result has not been confirmed in other studies.

#### 3.3.6. Effects of Tooth Brushing on Atrial Fibrillation

Two studies reported the effect of tooth brushing frequency on the occurrence of atrial fibrillation [13, 19], of which a cross-sectional study by Struppek [19] reported that the incidence of atrial fibrillation was 25%, 7.53%, and 7.12% in patients with different tooth brushing frequencies of once a week, once a day, and twice a day, respectively, and the advantage of ≥2/d tooth brushing to reduce the occurrence risk of atrial fibrillation was more significant in people aged ≥65 years. A retrospective cohort study by Chang [13] reported that the incidence of atrial fibrillation was 3.99%, 3.22%, and 2.51% in patients with different tooth brushing frequencies of 0-1/d, 2/d, and 3/d, respectively. Univariate and multivariate analyses showed that brushing frequency ≥3 times per day significantly reduced the risk of new atrial fibrillation (adjusted HR 0.90, 95% CI 0.83–0.98).

#### 3.3.7. Heterogeneity Test and Publication Bias

Due to the high heterogeneity of the included literature, a systematic review was conducted to describe the abovementioned outcome indicators in detail, without summary analysis for every outcome indicators. The reasons for the high heterogeneity were as follows: there were few included studies under each outcome indicator, with different designs of studies included case-control studies, retrospective cohort studies, prospective cohort studies, and cross-sectional studies, large span of publication years, differences in sample size, differences in population characteristics, and different definitions of outcome indicators (included only new-onset atrial fibrillation or new-onset atrial fibrillation and atrial flutter). The effect size was different (including HR/OR/RR after univariate/multivariate analysis) and the follow-up time was different. Sensitivity analysis, metaregression, and subgroup analysis were not performed. According to the NOS scale, the quality of cohort studies included in the present study was high. However, due to the small number of included studies and high heterogeneity under each outcome indicator, publication bias was not carried out, which may affect the results about the relationship between oral inflammatory diseases and atrial fibrillation.

## 4. Discussion

The purpose of this study was to evaluate the association between oral health and atrial fibrillation and evaluating the impact of regular oral care or tooth brushing on the occurrence risk of atrial fibrillation, as well as a systematic review of previous studies. The studies included in this systematic review included eight clinical studies with a total of 4,328,355 patients, of which five studies were large retrospective cohort studies [13–17], one was a prospective cohort study [12], one was a case-control study [18], and one was a cross-sectional study [19].

### 4.1. Relationship between PD, Oral Inflammatory Diseases, and Atrial Fibrillation

Previous studies have shown that the relationship between oral inflammatory diseases such as PD and atrial fibrillation is inconclusive. Aoyama et al. [20] found that the detection rate of *P. gingivalis* in atrial fibrillation patients aged 71∼90 years was significantly higher than that in patients with bradyarrhythmia. Miyauchi et al. [21] found that serum anti-*P. gingivalis* antibody type IV was an independent predictor of atrial fibrillation recurrence after catheter ablation (OR 1.937, 95% CI 1.301–2.884, and *P*=0.002). The abovementioned two studies [20, 21] indirectly suggested that oral inflammatory diseases such as PD may promote the occurrence, development, and recurrence of atrial fibrillation. Holm-Pedersen et al. [22] found that patients with one to two active coronal caries lesions had 2.8 times higher odds (95% CI 1.1–7.0) of arrhythmia than those without active coronal caries, but there was no association between arrhythmia and periodontal disease. And this study did not further analyze the relationship between active coronal caries, periodontitis, and atrial fibrillation. In the recent years, a number of large retrospective cohort studies and prospective cohort studies have suggested that oral inflammatory diseases such as periodontitis may be associated with an increased risk of atrial fibrillation, but the causal relationship still needs to be further verified. This study did not conduct a pooled analysis about the relationship between oral inflammatory diseases and atrial fibrillation because few studies could be included, with a large span of publication years, large differences in sample size, different design (included retrospective cohort study, prospective cohort study, and cross-sectional study), different outcome indicators (only recurrence of atrial fibrillation and recurrence of atrial fibrillation/atrial flutter), adjusted confounding factors, and varying follow-up time [12–19]. In addition, different characteristics of the study population may also affect the results of the study. For example, the prevalence rate of new-onset atrial fibrillation of male patients may be higher than that of female patients, and the prevalence rate of the elderly patients is significantly higher [18]. The differences of underlying diseases may also affect the results, such as heart failure, diabetes, chronic kidney disease, and coronary artery disease.

A Mendelian randomization study, which intended to verify the causal relationship between PD and cardiovascular diseases, such as atrial fibrillation, showed that there was no causal relationship between dental caries, PD, and cardiovascular diseases such as atrial fibrillation, and the correlation between PD and atrial fibrillation may be related to the common etiological pathway in the investigation study [23]. PD shared the same genetic and environmental risk factors with cardiovascular diseases such as atrial fibrillation and hypertension [24]. Although confounding factors were adjusted in the abovementioned studies, it cannot be ruled out that other potential confounding factors may have influenced the study results. Genetic polymorphisms, a possible confounding factor that is very difficult to control for, could increase the predisposition to atrial fibrillation and other cardiovascular diseases [25, 26]. Combined with the current research results, it is not easy to conclude whether PD and other oral inflammatory diseases affect the risk of new-onset atrial fibrillation. Future research is still needed to clarify the potential confounding factors between PD and atrial fibrillation, and more rigorous longitudinal studies will be needed to evaluate the association and specific causal relationship between oral inflammatory diseases such as PD and new-onset atrial fibrillation.

Sen et al. [12] revealed that severe PD significantly increased the occurrence risk of atrial fibrillation (RR = 1.31, 95% CI 1.06–1.62, *P*=0.01). This finding was supported by the results of Struppek et al. [19], which also found that the atrial fibrillation incidence in male was higher than in female. Only one prospective cohort study and one cross-sectional study elaborated the relationship between severe PD and atrial fibrillation in this review. However, considering that approximately 10% of the global population suffers from severe PD [8], approximately 16.3% of severe PD is associated with atrial fibrillation [12]. Therefore, more attention should be given to the occurrence risk of atrial fibrillation with severe PD. In the future, more large prospective studies are needed to evaluate the impact of different degrees of PD and other oral inflammatory diseases on the occurrence risk of atrial fibrillation. In addition, a cross-sectional study [18] suggested that the prevalence of new-onset atrial fibrillation in PD patients increases with age, and male patients at the same age were more likely to develop atrial fibrillation. Advanced research will be needed to evaluate the effects of severe PD on atrial fibrillation in different ages and sexes. In clinical practice, elderly male patients with severe PD should be considered.

### 4.2. Effects of Tooth Brushing and Oral Care on Atrial Fibrillation

Other important findings were that compared with long-term nonbrushing or tooth brushing <3/d, long-term ≥3/d tooth brushing significantly improved the occurrence risk of atrial fibrillation; compared with no oral hygiene or occasional oral hygiene, regular dental cleanings or oral care ≥1/year could significantly reduce the occurrence risk of atrial fibrillation. Although the current studies have not confirmed the correlation and causality between oral health and atrial fibrillation, the positive effect of oral care in reducing the risk of atrial fibrillation suggests that a better oral environment appears to be beneficial in improving the occurrence of atrial fibrillation. The current study found the advantages of tooth brushing and oral care in improving new-onset atrial fibrillation but failed to confirm whether brushing frequency ≥3 times per day and oral hygiene at different frequencies had inconsistent effects on new-onset atrial fibrillation. In addition, Chen et al. [14] reported that dental cleanings 0–2/year were a protective factor for atrial fibrillation compared with patients without oral care (HR 0.39, 95% CI 0.38–0.41), but dental cleanings >2/year were a risk factor for atrial fibrillation (HR 6.06, 95% CI 5.38–6.83). This may be associated with preexisting periodontitis in patients with dental cleanings >2/year or recall bias in self-report questionnaires [27]. Abovementioned results may suggest that proper oral hygiene appears to be important in improving the risk of atrial fibrillation, but the findings still need to be further validated in future studies, and more rigorous large-scale prospective studies are needed to explore the relationship between different frequency of oral care and the incidence of atrial fibrillation.

In addition, Omori et al. [28] showed that in hospitalized patients with heart disease complicated with periodontitis, six-step oral hygiene enhancement can improve the occurrence of atrial fibrillation after cardiac surgery. All of the abovementioned studies [12–14, 16, 19, 28] suggested that regular tooth brushing and oral care had an advantage in improving the occurrence and recurrence of atrial fibrillation. Therefore, the importance of long-term tooth brushing and oral hygiene should be emphasized in clinical practice and health promotion. At the same time, more studies are needed in the future to confirm the impact of different tooth brushing frequencies or oral care on the occurrence and recurrence of atrial fibrillation and whether high-frequency oral care increases the risk of atrial fibrillation.

### 4.3. Conjecture about Related Underlying Mechanisms

Although the causal relationship between PD and atrial fibrillation remains unclear, studies have shown that oral pathogens and inflammatory mediators may increase the occurrence risk and recurrence risk of atrial fibrillation, and the underlying mechanisms are as follows: First, regarding the role of oral pathogens, the oral cavity is a reservoir for many kinds of bacteria and microorganisms, and *P. gingivalis* can release lipopolysaccharide, downregulate the expression of L-type calcium channels in cardiomyocytes, and shorten the atrial effective refractory period [29]. *P. gingivalis* can increase the expression of toll-like receptor 2 (TLR-2) [30] and toll-like receptor 4 (TLR-4) [31], which can induce atrial fibrosis [32], reduce the transient outward potassium ion current [33], promote atrial structural remodeling and electrical remodeling, and increase the occurrence risk and recurrence risk of atrial fibrillation. Aggregator actinomycetes could promote inflammatory cell infiltration and induce cardiac remodeling, which may play an important role in increasing the occurrence risk of atrial fibrillation [34]. Second, regarding the roles of inflammatory mediators and systemic inflammation, periodontal disease could cause a persistent inflammatory response or bacteremia, release inflammatory mediators such as C-reactive protein and interleukin-6, stimulate myocardial cell hypertrophy and apoptosis, promote myocardial fibrosis, and shorten the atrial effective refractory period, which could increase the occurrence risk and recurrence risk of atrial fibrillation [35]. As the main virulence factor of *P. gingivalis*, fimbriae can regulate bacterial adhesion and invasion and stimulate a long-term chronic inflammatory response, inhibit interleukin-12 expression, and reduce the host's ability to clear inflammation [36]. Third, regarding immune response, periodontal pathogens can stimulate the release of large amounts of matrix metalloproteinases (MMPs) from lymphocytes and promote the occurrence of cardiovascular diseases [37]. In addition, antibodies against heat shock protein 60 (HSP-60) expressed by periodontal pathogens can cross-react with hSP-60 in the host body to activate T cells, leading to endothelial damage and atherosclerotic plaque formation and mediating cardiovascular disease [38]. However, whether MMPs or HSP-60-related immune responses of periodontal pathogens play important roles in atrial fibrillation remains unclear.

Regular tooth brushing and oral hygiene could reduce the colonization and accumulation of pathogenic bacteria, reduce the levels of C-reactive protein, interleukin-6, and other inflammatory mediators, and reduce the systemic inflammatory response [39], which may play important roles in preventing the occurrence and development of atrial fibrillation. Some studies have shown that periodontal therapy can alleviate elevated blood pressure, decrease white blood cell count and oxidative stress response, and reduce the expression of MMPs [40]. In addition, periodontal treatment can reduce serum total cholesterol, low-density lipoprotein, oxidized low-density lipoprotein, and other lipid levels [23]. The role of these mechanisms in preventing the occurrence and progression of atrial fibrillation remains unclear.

### 4.4. Summary and Outlook

At present, considering the high recurrence rate and the high risk of thromboembolism after the occurrence of atrial fibrillation, the guidelines for atrial fibrillation gradually focus on the primary prevention of atrial fibrillation. To evaluate the effects of different degrees of PD, oral hygiene, and tooth brushing on the risk of atrial fibrillation, this study included clinical studies related to oral inflammatory diseases such as PD, oral care, and atrial fibrillation. Eight clinical trials were included based on multiple database searches, and studies have shown that PD and other poor oral health condition may increase the risk of atrial fibrillation, of which severe PD has the highest risk, but the correlation and causality remained to be further verified. Patients with better oral health seemed to have a positive impact on preventing AF or reducing AF recurrence. Regular and moderate oral care and tooth brushing 2–3 times per day are effective measures for improving oral health and preventing AF. Oral health and oral disease prevention should be an important part of preventing new-onset atrial fibrillation. It is recommended to strengthen oral health publicity in future atrial fibrillation-related education.

### 4.5. Limitations

The limitations of this study are as follows: (1) Most included studies were retrospective cohort studies and cross-sectional studies, and the strength of the causal relationship was limited. (2) The number of included studies was limited, pooled analysis was not conducted due to the heterogeneity of the studies, and the validation strength was limited. (3) The correlation between tooth brushing frequencies of ≥3/d and higher and atrial fibrillation has not been determined. (4) The relationship between oral care ≥2/year and atrial fibrillation remains uncertain. (5) The relationship between antibiotic prevention of periodontal infection and atrial fibrillation is not clear. (6) The relationship between gargling or other forms of oral care and atrial fibrillation was also not included in the analysis.

## 5. Conclusion

Regular and moderate oral care, moderate frequent tooth brushing, and prevention of PD and other oral inflammatory diseases may be beneficial in reducing the occurrence and recurrence of atrial fibrillation. It is recommended to strengthen the popularization of oral health knowledge in the publicity related to atrial fibrillation.

## Figures and Tables

**Table 1:** General characteristics of the included studies.

Studies	Study design	Country	Follow-up	Sample size	Main characteristics of the included population	Exposure and nonexposure	Outcome indicator	Research result
Sen et al. [12]	A large prospective cohort study (ARIC)	United States of America	17 years	5,958	Patients without AF	Periodontal health, mild PD, moderate PD, and severe PD	Risk of new-onset AF	After adjusting for confounding factors, only severe PD increased the risk of AF Regular dental care reduced the risk of AF
						With or without dental care		

Chang et al. [13]	A nationwide, population-based, retrospective cohort (NHIS-HEALS)	Korea	10.5 years	161,286	Patients without AF or heart failure	With or without PD	Occurrence risk of AF and heart failure	After adjustment for confounders, PD did not increase the risk of atrial fibrillation or heart failure
						With or without dental visit for any reason		
						Frequency of tooth brushing		Missing teeth can increase the risk of heart failure
						Frequency of tooth brushing (0-1/d, 2/d, 3+/d);		
						With or without professional dental cleaning		Tooth brushing ≥3/d significantly reduced the risk of atrial fibrillation and heart failure occurrence

Chen et al. [14]	A nationwide, population-based, retrospective cohort (NHIRD)	Taiwan	4,075,682/3,405,292 person-years of follow-up	787,490	Patients without AF or atrial flutter	With or without PD. 0–2/year dental scaling or >2/year dental scaling	Risk of AF or atrial flutter	PD was a risk factor for atrial fibrillation or flutter
								Dental cleanings 0–2/year were a protective factor for atrial fibrillation or flutter
								Dental cleanings >2/year were a risk factor for atrial fibrillation or flutter

Hassan et al. [15]	A nationwide, population-based, retrospective cohort	France	4.8 ± 1.7 years	3,056,291	Patients without AF	With or without dental periapical abscess	Risk of new-onset AF	Periapical abscess had a low incidence of atrial fibrillation (10.69% *vs* 9.27%)
								Periapical abscess was an independent predictor of new-onset atrial fibrillation after adjustment for confounders
								The CHA2DS2VASc score in patients with periapical abscess had a predictive value for the development of AF

Chen et al. [16]	A nationwide, population-based, retrospective cohort (NHIRD)	Taiwan	4.6 ± 1.1 years	28,909	Patients without AF	With or without ≥1/year dental scaling	Risk of new-onset AF	After adjusting for confounders, dental scaling was associated with a reduced risk of AF

Hsu et al. [17]	A nationwide, population-based, retrospective cohort (NHIRD)	Taiwan	≥5 years	282,560	Patients without stroke and AF	With or without PD	Stroke incidence;	The incidence of stroke was 2.14-fold higher in the PD cohort than in the non-PD cohort; Patients with PD were 1.39 times more likely to have AF than those without PD
							Incidence of AF;	
							Incidence of atherosclerosis	

Im et al. [18]	Case-control study	Korea	18 months	227	Patients with AF	With or without presence of PD	Major adverse cardiac events (MACEs); any arrhythmic events	PD representative of chronic inflammation was an independent predictor of arrhythmic events and MACEs in patients with AF

Struppek et al. [19]	Cross-sectional study	Germany (Hamburg)	—	5,634	Patients without AF	No/mild PD, moderate PD, and severe PD;	Prevalence of AF	PD, IL-6, and CRP had no significant relationship with AF
						Never, 1/w, 1/d, 2/d tooth brushing		Tooth brushing ≥2/d had an association with a lower prevalence of AF

**Table 2:** Details about each risk of quality evaluation for each included cohort studies.

Studies	Representativeness of the exposed cohort	Selection of the nonexposed cohort	Ascertainment of exposure	Demonstration that the outcome of interest was not present at the start of the study	Comparability of cohorts on the basis of the design or analysis	Assessment of outcome	Sufficiently long follow-upfor outcomes to occur	Adequacy of cohort follow-up	Quality score
Chen et al. [16]	1	1	1	1	2	1	1	1	9
Chen et al. [14]	1	1	1	1	2	0	1	1	8
Chang et al. [13]	1	1	1	1	0	1	1	1	7
Sen et al. [12]	1	1	1	1	2	1	1	1	9
Hassan et al. [15]	1	1	1	1	0	1	1	1	7
Hsu et al. [17]	1	1	1	1	1	1	1	1	8

## Data Availability

The original contributions presented in the study are included within the article in the Supplementary Material section (Supplementary Table S1: Retrieval strategies used to filter literatures in Medline, Embase, Web of Science, and Cochrane Library), and further data can be obtained from the corresponding author upon request.

## Supplementary Materials

Supplementary Materials Supplementary Table S1: Retrieval strategies used to filter literatures in Medline, Embase, Web of Science, and Cochrane Library.
