# Supplementary material for: Effects of Oral Inflammatory Diseases and Oral Hygiene on Atrial Fibrillation: A Systematic Review
Source: Int J Clin Pract. 2023 Mar 27;2023:1750981. doi: 10.1155/2023/1750981 (PMC10070027; doi:10.1155/2023/1750981)
Supplement: Supplementary Materials — Supplementary Table S1: Retrieval strategies used to filter literatures in Medline, Embase, Web of Science, and Cochrane Library. [file 1750981.f1.docx]

**Supplementary Table S1.** Retrieval strategies used to filter literatures in Medline, Embase, Web of Science, and Cochrane

Library.

| **Database** | **Retrieval Strategies** | **Result** |
| --- | --- | --- |
| 1.PUBMED | #1：("Atrial Fibrillation”[MeSH Terms] OR "atrial Arrhythmia”[Title/Abstract] OR "AF”[Title/Abstract] OR "Atrial Fibrillations”[Title/Abstract] OR "Fibrillation, Atrial”[Title/Abstract] OR "Fibrillations, Atrial”[Title/Abstract] OR "Auricular Fibrillation”[Title/Abstract] OR "Auricular Fibrillations”[Title/Abstract] OR "Fibrillation, Auricular”[Title/Abstract] OR "Fibrillations, Auricular”[Title/Abstract] OR "Persistent Atrial Fibrillation”[Title/Abstract] OR "Atrial Fibrillation, Persistent”[Title/Abstract] OR "Atrial Fibrillations, Persistent”[Title/Abstract] OR "Fibrillation, Persistent Atrial”[Title/Abstract] OR "Fibrillations, Persistent Atrial”[Title/Abstract] OR "Persistent Atrial Fibrillations”[Title/Abstract] OR "Familial Atrial Fibrillation”[Title/Abstract] OR "Atrial Fibrillation, Familial”[Title/Abstract] OR "Atrial Fibrillations, Familial”[Title/Abstract] OR "Familial Atrial Fibrillations”[Title/Abstract] OR "Fibrillation, Familial Atrial”[Title/Abstract] OR "Fibrillations, Familial Atrial”[Title/Abstract] OR "Paroxysmal Atrial Fibrillation”[Title/Abstract] OR "Atrial Fibrillation, Paroxysmal”[Title/Abstract] OR "Atrial Fibrillations, Paroxysmal”[Title/Abstract] OR "Fibrillation, Paroxysmal Atrial”[Title/Abstract] OR "Fibrillations, Paroxysmal Atrial”[Title/Abstract] OR "Paroxysmal Atrial Fibrillations”[Title/Abstract])  #2：("Periodontitis”[MeSH Terms] OR "Aggressive Periodontitis”[Title/Abstract] OR "Chronic Periodontitis”[Title/Abstract] OR "Periapical Periodontitis”[Title/Abstract] OR "Periapical Abscess”[Title/Abstract] OR "Periapical Granuloma”[Title/Abstract] OR "Periodontal Abscess”[Title/Abstract] OR "Periodontal Pocket”[Title/Abstract] OR "periodontal disease”[Title/Abstract] OR "Pulpitis”[Title/Abstract] OR "Pericoronitis”[Title/Abstract] OR "periapical”[Title/Abstract] OR "dental abscess”[Title/Abstract] OR "tooth abscess”[Title/Abstract] OR "endodontic abscess”[Title/Abstract] OR "pulpal abscess”[Title/Abstract] OR "apical abscess”[Title/Abstract] OR "periradicular abscess”[Title/Abstract] OR "radicular abscess”[Title/Abstract] OR "acute dental infection”[Title/Abstract])  #3：#1 AND #2 | 225 |
| 2.EMBASE | #1：'Atrial Fibrillation':ti,ab,kw OR 'Atrial Arrhythmia':ti,ab,kw OR 'AF':ti,ab,kw OR 'Atrial Fibrillations':ti,ab,kw OR 'Fibrillation, Atrial':ti,ab,kw OR 'Fibrillations, Atrial':ti,ab,kw OR 'Auricular Fibrillation':ti,ab,kw OR 'Auricular Fibrillations':ti,ab,kw OR 'Fibrillation, Auricular':ti,ab,kw OR 'Fibrillations, Auricular':ti,ab,kw OR 'Persistent Atrial Fibrillation':ti,ab,kw OR 'Atrial Fibrillation, Persistent':ti,ab,kw OR 'Atrial Fibrillations, Persistent':ti,ab,kw OR 'Fibrillation, Persistent Atrial':ti,ab,kw OR 'Fibrillations, Persistent Atrial':ti,ab,kw OR 'Persistent Atrial Fibrillations':ti,ab,kw OR 'Familial Atrial Fibrillation':ti,ab,kw OR 'Atrial Fibrillation, Familial':ti,ab,kw OR 'Atrial Fibrillations, Familial':ti,ab,kw OR 'Familial Atrial Fibrillations':ti,ab,kw OR 'Fibrillation, Familial Atrial':ti,ab,kw OR 'Fibrillations, Familial Atrial':ti,ab,kw OR 'Paroxysmal Atrial Fibrillation':ti,ab,kw OR 'Atrial Fibrillation, Paroxysmal':ti,ab,kw OR 'Atrial Fibrillations, Paroxysmal':ti,ab,kw OR 'Fibrillation, Paroxysmal Atrial':ti,ab,kw OR 'Fibrillations, Paroxysmal Atrial':ti,ab,kw OR 'Paroxysmal Atrial Fibrillations':ti,ab,kw  #2：'Atrial Fibrillation'/exp  #3：'Periodontitis':ti,ab,kw OR 'Aggressive Periodontitis':ti,ab,kw OR 'Chronic Periodontitis':ti,ab,kw OR 'Periapical Periodontitis':ti,ab,kw OR 'Periapical Abscess':ti,ab,kw OR 'Periapical Granuloma':ti,ab,kw OR 'Periodontal Abscess':ti,ab,kw OR 'Periodontal Pocket':ti,ab,kw OR 'periodontal disease':ti,ab,kw OR 'Pulpitis':ti,ab,kw OR 'Pericoronitis':ti,ab,kw OR 'periapical':ti,ab,kw OR 'dental abscess':ti,ab,kw OR 'tooth abscess':ti,ab,kw OR 'endodontic abscess':ti,ab,kw OR 'pulpal abscess':ti,ab,kw OR 'apical abscess':ti,ab,kw OR 'periradicular abscess':ti,ab,kw OR 'radicular abscess':ti,ab,kw OR 'acute dental infection':ti,ab,kw  #4：'Periodontitis'/exp  #5：#1 OR #2  #6：#3 OR #4  #7：#5 AND #6 | 150 |
| 3.The Cochrane Library | #1：((Atrial Fibrillation) OR (atrial Arrhythmia) OR (AF) OR (Atrial Fibrillations) OR (Fibrillation, Atrial) OR (Fibrillations, Atrial) OR (Auricular Fibrillation) OR (Auricular Fibrillations) OR (Fibrillation, Auricular) OR (Fibrillations, Auricular) OR (Persistent Atrial Fibrillation) OR (Atrial Fibrillation, Persistent) OR (Atrial Fibrillations, Persistent) OR (Fibrillation, Persistent Atrial) OR (Fibrillations, Persistent Atrial) OR (Persistent Atrial Fibrillations) OR (Familial Atrial Fibrillation) OR (Atrial Fibrillation, Familial) OR (Atrial Fibrillations, Familial) OR (Familial Atrial Fibrillations) OR (Fibrillation, Familial Atrial) OR (Fibrillations, Familial Atrial) OR (Paroxysmal Atrial Fibrillation) OR (Atrial Fibrillation, Paroxysmal) OR (Atrial Fibrillations, Paroxysmal) OR (Fibrillation, Paroxysmal Atrial) OR (Fibrillations, Paroxysmal Atrial) OR (Paroxysmal Atrial Fibrillations)):ti,ab,kw  #2：MeSH descriptor: [Atrial Fibrillation] explode all trees  #3： ((Periodontitis) OR (Aggressive Periodontitis) OR (Chronic Periodontitis) OR (Periapical Periodontitis) OR (Periapical Abscess) OR (Periapical Granuloma)OR (Periodontal Abscess) OR (Periodontal Pocket) OR (periodontal disease)OR(Pulpitis) OR (Pericoronitis) OR (periapical) OR (dental abscess) OR (tooth abscess) OR (endodontic abscess) OR (pulpal abscess) OR (apical abscess) OR (periradicular abscess) OR (radicular abscess) OR (acute dental infection)):ti,ab,kw  #4: MeSH descriptor: [Psoriasis] explode all trees  #5: #1 OR #2  #6: #3 OR #4  #7: #5 AND #6 | 41 |
| 4.SCI-Web of Science | #1：("Atrial Fibrillation”[MeSH Terms] OR "atrial Arrhythmia”[Title/Abstract] OR "AF”[Title/Abstract] OR "Atrial Fibrillations”[Title/Abstract] OR "Fibrillation, Atrial”[Title/Abstract] OR "Fibrillations, Atrial”[Title/Abstract] OR "Auricular Fibrillation”[Title/Abstract] OR "Auricular Fibrillations”[Title/Abstract] OR "Fibrillation, Auricular”[Title/Abstract] OR "Fibrillations, Auricular”[Title/Abstract] OR "Persistent Atrial Fibrillation”[Title/Abstract] OR "Atrial Fibrillation, Persistent”[Title/Abstract] OR "Atrial Fibrillations, Persistent”[Title/Abstract] OR "Fibrillation, Persistent Atrial”[Title/Abstract] OR "Fibrillations, Persistent Atrial”[Title/Abstract] OR "Persistent Atrial Fibrillations”[Title/Abstract] OR "Familial Atrial Fibrillation”[Title/Abstract] OR "Atrial Fibrillation, Familial”[Title/Abstract] OR "Atrial Fibrillations, Familial”[Title/Abstract] OR "Familial Atrial Fibrillations”[Title/Abstract] OR "Fibrillation, Familial Atrial”[Title/Abstract] OR "Fibrillations, Familial Atrial”[Title/Abstract] OR "Paroxysmal Atrial Fibrillation”[Title/Abstract] OR "Atrial Fibrillation, Paroxysmal”[Title/Abstract] OR "Atrial Fibrillations, Paroxysmal”[Title/Abstract] OR "Fibrillation, Paroxysmal Atrial”[Title/Abstract] OR "Fibrillations, Paroxysmal Atrial”[Title/Abstract] OR "Paroxysmal Atrial Fibrillations”[Title/Abstract])  #2：("Periodontitis”[MeSH Terms] OR "Aggressive Periodontitis”[Title/Abstract] OR "Chronic Periodontitis”[Title/Abstract] OR "Periapical Periodontitis”[Title/Abstract] OR "Periapical Abscess”[Title/Abstract] OR "Periapical Granuloma”[Title/Abstract] OR "Periodontal Abscess”[Title/Abstract] OR "Periodontal Pocket”[Title/Abstract] OR "periodontal disease”[Title/Abstract] OR "Pulpitis”[Title/Abstract] OR "Pericoronitis”[Title/Abstract] OR "periapical”[Title/Abstract] OR "dental abscess”[Title/Abstract] OR "tooth abscess”[Title/Abstract] OR "endodontic abscess”[Title/Abstract] OR "pulpal abscess”[Title/Abstract] OR "apical abscess”[Title/Abstract] OR "periradicular abscess”[Title/Abstract] OR "radicular abscess”[Title/Abstract] OR "acute dental infection”[Title/Abstract])  #3：#1 AND #2 | 63 |
